# Retrospective analysis of calving interval data from seasonal-calving pasture-based New Zealand dairy herds

**DOI:** 10.3168/jdsc.2025-0942

**Published:** 2026-02-19

**Authors:** Laura Beasley, Krispin Kannan, Greg Chambers

**Affiliations:** 1 Zoetis, Auckland, 1010, New Zealand; 2 Veterinaryfirst, Te Awamutu, 3800, New Zealand; 3 EpiVets, Te Awamutu, 3800, New Zealand

## Abstract

This retrospective cross-sectional study described calving intervals (CIV) for spring-calving New Zealand dairy farms, evaluated the effects of cow-related factors, and investigated potential associations between CIV and future reproductive performance. Individual cow data were extracted from a veterinary dairy herd database for farms with at least 2 yr of data between June 2019 and May 2023. For the CIV analysis, observations were excluded from male animals, with >1 recorded calving event for a cow in a season, without a CIV, if the cow was born after June 1, 2020, or if CIV was <300 d. A descriptive analysis of CIV data was carried out, then herds with alternative calving patterns, cows that calved outside spring, and cows that had 2 consecutive calvings that skipped one or more seasons (carry-over cows) were excluded from the CIV analysis. A mixed multivariable linear regression model was constructed to evaluate associations between CIV and potential explanatory variables (age, breed, dairy season, and milk yield). A separate dataset was constructed to evaluate effect of CIV on odds of future reproductive success (a recorded calving event in the subsequent spring calving season). The same inclusion criteria were applied as for the analysis of CIV, except cows with only 2 dairy seasons of data or born after June 1, 2019, were excluded but carry-over cows were included. A mixed multivariable generalized linear regression model was constructed to evaluate associations between odds of reproductive success and the potential explanatory variables of previous CIV, age, breed, season, and mean milk solids yield. Mean (SD) CIV was 368.8 (23.96) d for spring-calving herds, compared with 381.9 (53.6) d for herds with alternative calving patterns. Calving intervals were longer for herds that carried some cows over between seasons (mean [SD] 376.3 [58.3] d) compared with herds that did not (mean [SD] 366 [23] d). The regression model confirmed that mean CIV varied between seasons, was shorter for cows with higher milk production, and the interval between first and second calvings was 7 d longer than the interval between subsequent calvings. Most variation in CIV occurred within the herd at the cow-season level. Probability of reproductive success (adjusted for age, season, and milk production) declined with CIV. When averaged across farms, age groups, seasons, and milk production, the predicted probability of a successful mating declined from 0.69 (95% CI = 0.66–0.72) at a CIV of 357 d, to 0.05 (95% CI = 0.03–0.07) at 538 d. Calving intervals could potentially be used to identify animals at higher risk of reproductive failure within seasonal-calving dairy herds. However, causal inferences are limited by the retrospective study design and limited information available. In seasonal-calving systems, CIV is closely linked to calving date, so prospective studies explicitly adjusting for calving date are warranted and we interpret CIV primarily as a timing-influenced, operational indicator that complements calving date for within-herd screening rather than a standalone causal determinant.

## Highlights

• Cows in spring-calving, pasture-based dairy herds calved every 369 days on average.
• There was little variation in average calving interval between herds.
• The interval between calving events was around 7 days longer for young cows.
• Cows producing more milk had slightly shorter gaps between calving events.
• A longer interval between calvings lowered the odds of the cow calving again next year.

## Graphical Abstract

Historical cow data were extracted and analyzed for 161,923 cows from 265 New Zealand spring-calving, pasture-based dairy herds; the average interval between 2 calving events for the same cow was 369 days. The gap between first and second calving events was, on average, 7 days longer compared with the gap between subsequent calvings, and cows with higher milk production had slightly shorter intervals between calving events. There was little variation in average calving interval between herds, but more variation between cows within the same herd and between different dairy seasons. A smaller dataset was used (108,430 cows from 206 farms) to examine whether the interval between 2 calving events affected the odds of the cow having a future calving event recorded. A longer gap between calving events resulted in a lower chance of the cow calving again the following season.

**Figure:**
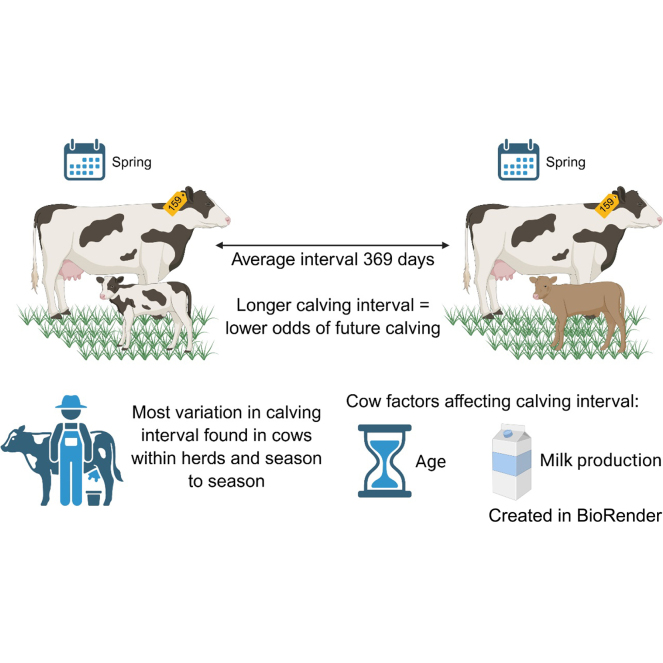

Calving interval (**CIV**) is the number of days between one calving event and the subsequent calving event for the same cow. In nonseasonal dairy systems, CIV is a commonly used reproductive performance indicator because it is associated with the interval between parturition and re-conception, and therefore reproductive efficiency (Armengol et al., 2022). However, CIV has limitations as a reproductive performance indicator because CIV excludes cows that do not calve again.

Pasture-based dairy systems match feed demand (linked to milk production) with grass growth by operating a spring seasonal-calving system (Roche et al., 2017). In these seasonal-calving systems, CIV and calving date are mechanically coupled: Later calving shortens the postpartum interval before a fixed mating start date, which can adversely affect subsequent fertility (Moore et al., 2021). Cows with a longer CIV may therefore be less likely to calve again in the subsequent season, but no published data are available to support or refute this hypothesis. Calving interval is seldom used in pasture-based dairy herds (Morton, 2010). It may have value as a practical, within-herd screening indicator to flag cows at elevated risk of poor reproductive performance; however, calving date is essential for causal interpretation. Because the end of lactation is usually governed by management factors such as feed availability, a CIV longer than 365 d will lead to a shorter subsequent lactation. This may potentially result in lower total milk production for the subsequent lactation, but the true impact on milk production will depend on lactation characteristics.

Despite widespread use of this performance indicator in housed dairy farming systems, there have been relatively few data published examining CIV in seasonal-calving, pasture-based dairy herds. Reported mean CIV for seasonal-calving herds ranges between 364 and 398 d (Cunningham et al., 1976; Olori et al., 2002). Evans et al. (2006) reported that proportion of North American Holstein Friesian (**NAHF**) genetics, age at first, second, and third calving, and calving date were associated with CIV for cows in Irish dairy herds, but this is the only analysis examining association between breed and CIV for seasonal-calving, pasture-based dairy herds.

Published CIV data from New Zealand dairy herds were collected before 2015 (Macmillan and Moller, 1977; Grosshans et al., 1997), after which routine induction of parturition (**RIoP**) could no longer be practiced as a tool to manage calving pattens (Verdon, 2021). Routine induction of parturition was common practice on New Zealand dairy farms from the 1970s onward, and as RIoP advances the calving date of late-calving cows, this practice may affect CIV data reported during this period (Macmillan, 2002).

This retrospective cross-sectional study examined 4 yr of reproductive data from New Zealand dairy farms under the care of Veterinaryfirst (Te Awamutu, New Zealand) between 2019 and 2023. Our objectives were to describe CIV for cows from pasture-based, spring-calving farms, to evaluate the influence of cow age, breed, dairy season, and milk production on CIV (CIV analysis), and to explore any potential association between CIV and future reproductive success (reproductive success analysis).

Data were sourced on December 5, 2023, from commercial New Zealand dairy farms that were serviced by one veterinary business (Veterinaryfirst). Dairy farming seasons were defined as the period between June 1 and May 31, and the data were extracted as one electronic spreadsheet (Excel, Microsoft) per farm per season via InfoVet (Zoetis, New Zealand). Raw data were imported into R Studio 4.2.2 for statistical analysis (R Core Team, 2022). The data were collated and merged by uniquely identifying cows on each farm, then examined for completeness, duplication, consistency, and spurious values.

Individual farms were classified as eligible for analysis if there were at least 2 seasons of data between June 1, 2019, and May 31, 2023, and partial or complete data for unique cow identification, individual cow calving dates, cow breed and date of birth, and herd test milk solids yield. Herds were excluded from both the CIV analysis and reproductive success analysis if >1% of calving events took place during the autumn (January to May inclusive). Observations were excluded if they were from males; if the cow had more than one unique recorded calving date for that season; the cow was born after June 1, 2020 (CIV analysis), or June 1, 2019 (reproductive success analysis); the CIV was <300 d (indicating either premature parturition or a recording error); there was no CIV; or the calving occurred during the autumn. In addition, observations were removed from the CIV analysis if they related to a carry-over event, defined as a cow having 2 consecutive calving events that skipped a season (Gardner et al., 2020).

All variables were described with tables and distribution plots and checked for completeness and spurious values. For a given season, the CIV was defined as the number of days between that season's calving event and the next season's calving event (if it occurred). This allowed the association between a given season's milk yield and CIV between that season and the next to be explored. Milk production was reported as mean herd test milk solids yield for the season, and milk solids values recorded as 0 kg were classified as missing data.

Breed for each cow was categorized into Ayrshire, Jersey, Friesian (≥12/16 of the respective breeds), Friesian-Jersey cross (contains only Friesian and Jersey genetics that sum to 16/16, and the Friesian and Jersey components are between 4/16 and 12/16 inclusive), and “other.” Herd-level mating program variables (e.g., planned start of mating, synchronization, use of short-gestation semen) were not available and therefore could not be included explicitly; between-herd and seasonal differences in mating management were accounted for via farm-level random effects and season terms. Herds were classified as having an alternative calving pattern if >1% of calving events took place during autumn (January to May inclusive). Descriptive CIV data (mean and SD) were reported for this dataset, with CIV described separately for herds classified as spring calving versus herds with alternative calving patterns, and for herds that carried over some cows versus herds that did not carry over any cows.

To evaluate associations between CIV and the potential explanatory variables of age, breed, season, and milk yield, a mixed multivariable linear regression model was constructed to provide adjusted associations. This analysis was restricted to cows with herd test milk solids yield data. Correlation of cows within farms was accounted for by a random intercept for farm, and serial correlation of seasons within cows was accounted for with a random intercept for cow and a compound symmetry covariance term (to account for the autocorrelation of repeated measures). All candidate explanatory variables were specified a priori based on biological plausibility and were included simultaneously in a multivariable mixed-effects regression model. Given the large sample size, no variable selection or elimination procedures were used, and inference was based on estimated effect sizes and associated uncertainty. The final model was tested for the assumptions of independence, linearity, homoscedasticity, and normally distributed residuals by evaluation of diagnostic plots, at the farm, cow, and test levels. The central limit theorem was invoked for the normality assumption, and the distribution of higher-level residuals was not critically explored because they do not influence fixed effects coefficients. Because of the large number of observations at each hierarchical level, influential observations were not explored because a single observation would not be expected to influence the model parameters. The intraclass correlation coefficient (**ICC**), a measure of correlation within groups, was calculated by building a null mixed regression model and obtaining the variance of the farm and cow random effects terms and the remaining cow-season-level variance. Although individual calving dates were available and used to derive CIV, calving date was not included as a covariate in these models.

Reproductive success was defined as a recorded calving event in the subsequent spring calving season. To test the null hypothesis that CIV was not associated with the odds of reproductive success, a mixed multivariable generalized linear regression model with a logit link was constructed to provide adjusted associations. Calving intervals without a corresponding reproductive success outcome were removed from analysis (e.g., cows with only 2 seasons of data, as CIV between seasons 1 and 2 could be calculated but reproductive success in season 3 could not be evaluated). This analysis was restricted to the dairy seasons between 2019 and 2022 because reproductive success could not be calculated for the 2022–2023 season in the absence of 2023–2024 season data, and to cows with complete herd test milk solids yield data. Cows that had carry-over events were included, with the absence of a calving event in the carried over season classified as a reproductive failure for the previous season. Potential explanatory variables were age, breed, season, mean milk solids yield, and CIV; the latter 2 variables were standardized to prevent model convergence issues. Correlation of cows within farms was accounted for by a random intercept for farm. Farms with no successes (n = 3 farms) or <100 observations (n = 3 farms), or both, were deleted to prevent model convergence problems due to complete separation. There was little variance at the cow level because each cow had only 1 or 2 observations each, with values of 0 or 1 (i.e., a mean of 0, 0.5, or 1), so a random intercept for cow was not included. Again, all candidate explanatory variables were specified a priori based on biological plausibility and were included simultaneously in a multivariable mixed-effects regression model. Polynomial terms were added for mean milk solids yield and CIV to address linearity violations. The final model was tested for the assumption of linearity with diagnostic plots. Influential farms were explored with deletion methods. Influential covariate patterns were explored by constructing an analogous fixed effects model that included farm. The ICC was calculated using the latent variable method (Wu et al., 2012). Population-average predicted probabilities of reproductive success were obtained from the final mixed-effects logistic regression model using the *emmeans* package (Lenth et al., 2024), by varying CIV over a vector of values and averaging predictions over the other fixed-effect covariates (with random effects set to zero).

The raw data contained a total of 2,220,026 observations from 539,801 unique animals on 368 farms. After collapsing herd test data to a single mean value per season, there were 1,014,974 observations from 539,801 unique animals on 368 farms. After exclusion criteria were applied, the dataset for the CIV analysis contained 283,688 CIV from 161,923 cows on 265 farms. Mean herd CIV was longer for herds with alternative calving patterns (n = 33 herds) than for spring calving–only herds (n = 265), with means (SD) of 381.9 (53.6) and 368.8 (24) d, respectively (*P* < 0.001). Including data from carry-over cows, CIV was longer among herds that recorded some carry-over calving events, with means (SD) of 376.3 (58.3) d and 366 (23) d for farms that did (n = 230 farms) and did not (n = 36 farms) record some carry-over calving events, respectively (*P* < 0.001). The difference in herd counts between analyses reflects differing exclusion criteria: One additional herd was included in the carry-over analysis because it contained a single cow with a valid CIV that was classified as a carry-over event and was therefore excluded from the main analysis but retained when carry-over events were considered.

The number of observations per farm ranged from 10 to 5,535, with a mean of 1,071. Cow seasons had a median (interquartile range) age of 4 (3–6) yr, and a mean (SD) season herd test milk solids yield of 1.65 (0.41) kg. The most common cow breed in this dataset was Friesian (n = 109,065 [38%]), followed by “other” (n = 83,075 [29%]), Friesian-Jersey (n = 66,279 [23%]), Jersey (n = 23,732 [8.4%]), and finally Ayrshire (n = 1,537 [0.5%]). Calving interval was approximately normally distributed, with a mean (SD) of 368.8 (23.96) d and range of 300 to 536 d. Between-farm variation in CIV was small (farm mean CIV 344.5–377.63 d); 35 farms had a mean CIV under 365 d.

After adjusting for other variables and including random effects for farm and cow, CIV was negatively associated with season mean herd test milk solids yield; CIV was predicted to be 0.29 d shorter for each 0.1 kg increase in yield. Also, CIV varied between seasons, and the interval between first and second calvings was 7.42 d longer than intervals between subsequent calvings (Table 1). The ICC was 0.101 at the farm level and 0.113 at the cow level. That means that 10%, 11%, and 79% of the variation in CIV occurred at the farm, cow, and cow-season levels, respectively; there was a large range within each farm but little range between farm means.

**Table 1:** Final multivariable mixed linear regression model of the association between calving interval and other variables, in a study of 250,069 calving intervals from 146,978 cows on 247 spring-calving, pasture-based New Zealand dairy farms

Variable	Coefficient (95% CI)	*P*-value
Intercept	378.24 (376.88 to 379.61)	
Cow age		<0.001
2 yr	Referent	
3+ yr	−7.42 (−7.67 to −7.17)	
Cow breed		0.837
Ayrshire	Referent	
Friesian	0.40 (−0.85 to 1.66)	
Friesian-Jersey cross	0.32 (−0.94 to 1.57)	
Jersey	0.26 (−1.02 to 1.55)	
Other	0.36 (−0.89 to 1.62)	
Season^1^		<0.001
2019–2020	Referent	
2020–2021	0.70 (0.44 to 0.96)	
2021–2022	−0.56 (−0.82 to −0.31)	
Season mean herd test milk solids yield (kg)	−2.94 (−3.21 to −2.66)	<0.001

1 Dairy farming seasons were defined as the period between June 1 and May 31.

For the reproductive success analysis, reproductive success outcomes were not available for 109,112 observations in the dataset used for testing associations between explanatory variables and CIV, which reduced the dataset to 181,330 observations from 120,365 cows on 224 farms. Overall, 123,637/181,330 (68.2%) of cow seasons led to reproductive success. Cow seasons had a median (interquartile range) age of 4 (3–6) yr and mean (SD) season herd test milk solids yield and CIV of 1.66 (0.42) kg and 369.1 (24.14) d, respectively. After exclusion criteria were applied, the dataset for the reproductive success analysis contained 158,573 observations from 108,430 cows on 206 farms. These deletions resulted in 5 farms having no successful reproductive outcomes, but they were retained in the model dataset.

Calving interval, cow age, breed, season, and season mean herd test milk solids yield were associated with the odds of reproductive success (Table 2). The associations with CIV and season mean herd test milk solids yield were nonlinear. The model included both linear and quadratic (squared) terms for standardized CIV; after adjusting for other variables and including a random effect for farm, the odds ratios were 0.89 (95% CI = 0.88–0.91) for a 1-SD increase in CIV (approximately 24 d) and 0.93 (95% CI = 0.92–0.93) for a 1-unit increase in squared standardized CIV. Population-average predicted probabilities of reproductive success were highest at a CIV of about 350 d, then declined increasingly steeply at longer intervals, from 0.69 (95% CI = 0.66–0.72) at 357 d to 0.05 (95% CI = 0.03–0.07) at 538 d (Figure 1). Two-year-old cows had approximately double the odds of reproductive success (i.e., a recorded calving event in the subsequent spring calving season) compared with older cows, and the odds were 21% lower in the 2020–2021 season than the 2019–2020 season. Season mean herd test milk solids yield had a curvilinear association with reproductive success, with the probability of success increasing with yield until a peak of approximately 2.0 kg, then declining again with higher yields. Because calving date was not included as a covariate, the observed association between CIV and reproductive success should be interpreted as a composite of effects related to both CIV and calving timing within the season, rather than as the isolated effect of CIV alone.

**Table 2:** Final multivariable mixed linear regression model of the association between reproductive success (having a recording calving event in the following season) and explanatory variables using data containing 158,573 observations from 108,430 cows on 206 spring-calving, pasture-based New Zealand dairy farms

Variable	Odds ratio (95% CI)	*P*-value
Intercept	4.82 (3.71 to 6.26)	
Cow age		<0.001
2 yr	Referent	
3+ yr	0.53 (0.51 to 0.55)	
Cow breed		0.005
Ayrshire	Referent	
Friesian	0.89 (0.72 to 1.10)	
Friesian-Jersey cross	0.93 (0.76 to 1.16)	
Jersey	0.85 (0.69 to 1.06)	
Other	0.90 (0.73 to 1.11)	
Season^1^ mean herd test milk solids yield (kg; standardized)	1.01 (0.99 to 1.03)	0.380
Season mean herd test milk solids yield (kg; standardized) squared	0.94 (0.93 to 0.95)	<0.001

1 Dairy farming seasons were defined as the period between June 1 and May 31.

**Figure 1:**
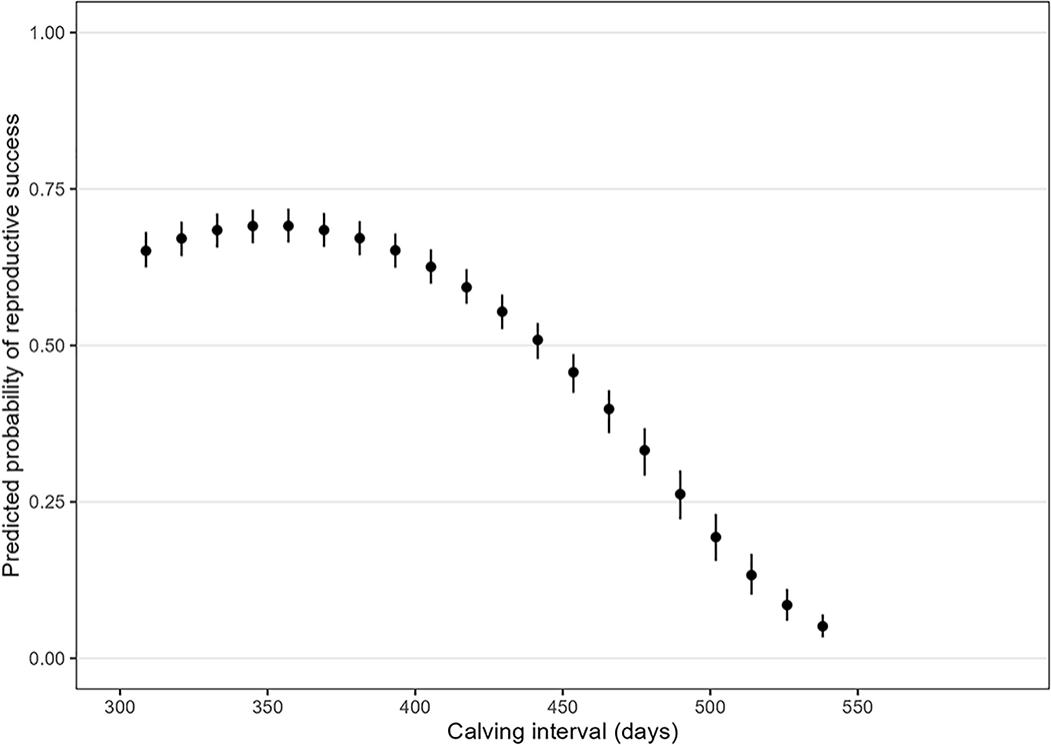
Population-average predicted probability of reproductive success (a calving event being recorded in the following season) predicted by a multivariable mixed generalized regression model for a cow with a range of calving intervals, margined across other variables, in an analysis of the association between calving interval and reproductive success of 158,573 observations from 108,430 cows on 206 New Zealand dairy farms. Vertical lines represent 95% CI.

Overall, mean CIV was just over one year at 369 d, ranging from 300 to 536 d, but this range was artificially imposed by exclusion criteria. Herd mean CIV from this dataset is similar to that reported by Macmillan and Moller (1977) for New Zealand dairy herds approximately 50 yr ago (mean CI 364 d), and to the mean CIV of 365.2 d reported by Grosshans et al. (1997) for New Zealand dairy cows in their second lactation. This does not support a negative impact on herd CIV following the phasing out of RIoP in 2015; however, this does not mean that RIoP had no impact on herd reproductive management. Average mating length for New Zealand dairy herds was recently reported as approximately 75 d (Jayawardana et al., 2022), roughly 4 wk shorter the average mating length of 14.6 wk reported by Macmillan and Moller (1977), which suggests that farms may have manipulated mating period length to maintain herd CIV.

In seasonal-calving herds, CIV and calving date are structurally linked. Later calving shortens the postpartum interval before the fixed mating start date and can reduce lactation length, which may adversely affect fertility and productivity. When calving date is not included as a covariate, associations between CIV and outcomes are likely to reflect this indirect timing pathway in addition to any direct effects of CIV. Our models did not include calving date among covariates, and unmeasured reproductive management (e.g., synchronization, use of short gestation length semen) may further confound both timing and CIV. Thus, although CIV can be operationally useful for within-herd screening, it should be interpreted alongside calving date rather than as a standalone predictor. Its utility lies in identifying cows with prolonged intervals who may merit targeted management, recognizing that some will be culled and thus excluded from CIV-based measures. Future prospective studies should explicitly incorporate calving date and detailed mating program records to separate indirect timing effects from any direct CIV effects.

Reported values of mean CIV for New Zealand dairy herds have been very consistent over a span of several decades, which contrasts with published data from the outwardly similar pasture-based seasonal dairy farming nation of Ireland. Over a 16-yr period, mean CIV for Irish dairy herds varied between 369 and 398 d (Olori et al., 2002; Evans et al., 2006), with mean CIV for New Zealand dairy herds falling at the lower end of this range. The underlying reasons for these differences are likely to be multifactorial and difficult to fully evaluate due to the retrospective and observational nature of the datasets. Differing breed composition between both individual and national dairy herds may be a contributing factor. Evans et al. (2006) reported a positive cow-level correlation between proportion of NAHF genetics and CIV, whereas the proportion of NAHF genetics in their dataset increased across the study period, reaching a high of 68%. This is substantially different from the breed composition for the New Zealand herds in this dataset, where most cows were either crossbred or non-Friesian dairy breeds.

In this dataset, after including a farm random effect, CIV was associated with age, season, and milk yield, but not breed, although breeds were classified differently and proportion of NAHF genetics was not recorded. Shorter CIV for Jersey cows compared with Friesians during their first and second lactations has been reported previously (Grosshans et al., 1997). In the present study, which included cows across all ages and adjusted for farm-level clustering and other covariates, breed differences in CIV were estimated with substantial uncertainty, and the data were consistent with a range of associations including small differences between breeds. The curvilinear relationship between milk yield and CIV indicates a complex association between production and fertility. Improved cow management (e.g., nutrition, health) may increase both milk yield and reproductive performance (Buckley et al., 2003), yet high genetic merit for production can be associated with poorer fertility (Pryce and Veerkamp, 2001).

Reproductive management will also affect CIV at both cow and herd level. New Zealand dairy farmers often choose to mate maiden heifers earlier than the milking herd to allow more time for recovery between first calving and the subsequent spring mating season. This is likely to have been reflected in the longer interval between first and second calvings compared with the interval between subsequent calvings found in this dataset (because the mating start date is typically the same for all age groups among lactating cows). Grosshans et al. (1997) reported that the interval between first and second calvings was 9.3 d longer compared with the interval between second and third calvings for New Zealand dairy cows. Although the direction of this effect is comparable with that found in this dataset, substantial differences in methodology between these 2 studies mean that these findings are only loosely comparable.

At herd level, management decisions on when to mate cows and whether to retain nonpregnant cows for subsequent mating are likely to affect CIV. Comparisons of CIV in herds with alternative calving patterns versus spring calving–only herds were descriptive and conducted on the full dataset before exclusions; the main CIV analysis then excluded carry-over events and calvings occurring outside the spring calving season because most New Zealand dairy cows are spring calving (Brownlie et al., 2014). However, a longer mean CIV of 384 d has been reported for “town-supply” New Zealand dairy herds. These are farms that supply milk throughout the year for local whole milk consumption and therefore need to calve cows outside the spring calving season (Fielden et al., 1980), illustrating the impact of farm management on CIV. This finding was replicated in this dataset, where mean CIV was longer for herds with alternative calving patterns and herds that recorded some carry-over calving events compared with herds that only recorded spring calving events, or herds than did not record any carry-over calving events. In split-calving herds or herds that allow carry-over calving events, farmers have the option to retain nonpregnant cows for mating in a subsequent season rather than culling them, thus producing longer CIV values for these cows.

We excluded known reproductive management factors (for example, non-spring or carry-over calving events), and adjusted for age, breed, season, farm, and milk yield, but there are many other unmeasured factors that may influence CIV, such as the use of short gestation length semen or synchrony programs. Some of these factors are swept up under the random effect for farm (the “herd effect”), and the reasons underlying each herd's reproductive management strategy were not documented.

Most of the variation within the current dataset occurred within herd at the cow-season level, as opposed to at the herd or cow levels, therefore knowing either the herd or the cow did not add a lot of value in predicting a cow's CIV between 2 seasons chosen at random. Calving interval appeared to fluctuate a lot between seasons, though this finding was limited by having only a small number of seasons per cow. These between-season CIV fluctuations may be related to differing weather; rainfall, daylight hours, and temperature affect growth, quality, and intake of the major feed source (grass) for pasture-based dairy herds. Nutrition before and during mating is a major determinant of reproductive performance in dairy cattle (Macmillan, 2002). A longer-term dataset may show less variance at the season level with more data per cow, but it is also important to consider that CIV data will only be available for cows that are retained in the herd to calve again.

We identified a negative association between CIV and likelihood of a recorded calving event in the subsequent season, showing that CIV needs to be considered along with cow survival data to avoid inadvertently excluding information from cows that did not calve again. At the cow level, longer CIV were associated with lower subsequent reproductive success; however, interpretation of CIV as a proactive risk indicator should be made with caution. In seasonal-calving systems, later-calving cows are more likely to receive synchronization or other reproductive interventions, which can shorten observed CIV despite underlying subfertility. The absence of detailed mating and synchronization data may therefore weaken observed associations, potentially biasing estimates toward the null. Presence of a recorded calving event in the following season was used as a measure of reproductive success, but cows may have been culled for other reasons not related to fertility (e.g., poor milk production, mastitis).

This study has successfully updated knowledge of CIV within New Zealand's seasonal-calving, pasture-based dairy system and revealed that most variation in CIV occurred within herds rather than between herds. A negative association was identified between CIV and likelihood of a subsequent recorded calving event, but this should only be taken as a weak estimate of reproductive success. Overall, CIV is best interpreted as a timing-influenced operational indicator that complements calving date for within-herd screening in seasonal herds, rather than a standalone predictor of fertility or productivity.
